# Structural brain effects of alcohol use patterns in alcohol use disorder: a systematic review and meta-analysis

**DOI:** 10.1192/j.eurpsy.2026.10506

**Published:** 2026-07-31

**Authors:** T. Bernabeu, S. Vollstädt‐Klein, F. Kiefer, B. Rolland

**Affiliations:** 1 PSYR2, CNRL, INSERM U1028, CNRS UMR5292, UCBL1, Bron; 2 Université Claude Bernard Lyon 1, Lyon; 3 Centre Hospitalier Le Vinatier, Bron; 4 Service Universitaire d’Addictologie de Lyon (SUAL), HCL, CH Le Vinatier, Lyon, France; 5 Department of Addictive Behaviour and Addiction Medicine, Central Institute of Mental Health, Medical Faculty Mannheim, University of Heidelberg; 6 Mannheim Center for Translational Neuroscience, Medical Faculty Mannheim, University of Heidelberg; 7 German Center for Mental Health (DZPG), partner site Mannheim‐Heidelberg‐Ulm, Mannheim; 8 Feuerlein Centre on Translational Addiction Medicine, University of Heidelberg, Heidelberg, Germany

## Abstract

**Introduction:**

Alcohol Use Disorder (AUD) affects over 14% of the population and contributes to millions of premature deaths worldwide. Chronic alcohol use is associated with structural brain alterations, particularly in the prefrontal cortex, striatum, thalamus, and cerebellum. However, the relationship between specific drinking patterns (e.g., duration, intake, age of onset) and neuroanatomical changes remains poorly understood.

**Objectives:**

This study aimed to identify consistent brain structural alterations in individuals with AUD and to explore dose-dependent effects of alcohol use patterns using a systematic review and meta-analysis.

**Methods:**

Following PRISMA 2020 guidelines, we conducted a systematic review and voxel-based morphometry (VBM) meta-analysis of 28 gray matter and 8 white matter studies. Studies were included if they compared AUD patients with healthy controls. Meta-analyses and meta-regressions were performed using SDM-PSI (v6.22), applying TFCE-FWE correction (p < 0.05).

**Results:**

Compared to controls, AUD patients showed significant gray matter reductions in the cingulate, insula, Heschl gyrus, and postcentral gyri. Higher AUDIT scores and longer use duration were associated with greater volume loss in frontal and cerebellar regions. Earlier age of onset predicted stronger deficits in prefrontal areas. Longer abstinence was linked to partial volume recovery in the corpus callosum. White matter studies revealed corpus callosum reductions and putamen changes associated with early alcohol initiation.

**Conclusions:**

AUD is linked to widespread gray and white matter reductions. Meta-regressions demonstrate dose-response relationships and suggest partial brain recovery with abstinence, underscoring the importance of early intervention.

**Disclosure of Interest:**

None Declared

